# Improving Safety and Effectiveness of Out-of-Hours Inpatient Medical Handover, by Developing a Rio-Embedded Handover Platform

**DOI:** 10.1192/bjo.2026.11436

**Published:** 2026-06-30

**Authors:** Katherine Macfarland, Senem Leveson, Hussain Moeed, Mariana Garcia

**Affiliations:** North London NHS Foundation Trust, London, United Kingdom

## Abstract

**Aims:**

To improve the safety, effectiveness and staff experience of inpatient medical handover to at least 70% satisfaction ratings.

The pre-existing medical handover processes varied significantly across the five boroughs within the North London NHS Foundation Trust (NLFT), leading to issues with poor accessibility, information governance, audit capability and user experience.

A new, unified, Trust-wide handover platform could tackle these issues and support consistent resident doctor processes and communication.

**Methods:**

Over successive phases, we developed and implemented more digitally mature handover platforms. In our final phase, this handover platform was in Rio, the Trust's existing Electronic Patient Record (EPR). Each phase contained multiple iterative PDSA cycles.

Phase 1: Initial Digital Handover Improvements (2021–2023)

Local QI projects at our two legacy Trusts (which later merged to form NLFT) sought to improve existing handover methods. One Trust introduced a Microsoft Teams list for handover.

Phase 2: Rio-Based Handover Pilot (May 2024–January 2025)

A working group was formed in May 2024, including the Trust’s Chief Clinical Information Officer (CCIO), RiO applications expert and three resident doctors. They collaboratively designed a new platform in RiO and a pilot was launched at a single inpatient site in January 2025.

Phase 3: RiO-Based Handover Full Rollout (January–June 2025)

In March 2025, a refined iteration of the RiO-based medical handover platform was rolled out across the remaining four inpatient sites in NLFT. Before rollout, stakeholder engagement sessions were held to train staff and promote awareness of the platform. After rollout, further sessions took place to gather informal feedback.

**Results:**

Formal staff survey feedback was gathered two months after Trust-wide implementation and showed significant improvement across all key measures. All measures are ratings out of 10.

Safe & Effective Handover (Old: 6.19 → New: 8.25), Ease of Access (6.25 → 8.44), User Experience (5.75 → 8.18), and Information Governance (4.70 → 8.56).

**Conclusion:**

Trust-wide implementation of the new Rio-embedded medical handover led to positive staff survey feedback. This highlighted significant improvements in safety, effectiveness, user experience and information governance. The collaboration betweenresident doctors, digital teams, and clinical leaders served as a model for future multi-disciplinary QI projects. Resident doctor leadership on stakeholder engagement sessions was particularly effective in embedding the new platform culturally.

Long-term, opportunities exist to integrate other handovers into Rio, such as the emergency system handover, to further enhance patient safety and operational efficiency.

